# Dataset of lithium phosphate recovery from a low concentrated lithium-containing solution

**DOI:** 10.1016/j.dib.2019.104044

**Published:** 2019-05-24

**Authors:** Chunlong Zhao, Yanling Zhang, Hongbin Cao, Xiaohong Zheng, Tom Van Gerven, Yingyan Hu, Zhi Sun

**Affiliations:** a Beijing Engineering Research Center of Process Pollution Control, National Engineering Laboratory for Hydrometallurgical Cleaner Production & Technology, Chinese Academy of Sciences, Beijing, 100190, China; b State Key Laboratory of Advanced Metallurgy, University of Science and Technology Beijing, Beijing, 100083, China; c Department of Chemical Engineering, KU Leuven, De Croylaan 46, B-3001, Leuven, Belgium; d School of Engineering and Technology, China University of Geosciences, Beijing, 100083, China

**Keywords:** Lithium phosphate, Recovery, Precipitation, Lithium-containing solution

## Abstract

The lithium-containing solution is also rich in lithium after preparation of lithium carbonate. With the depletion of primary lithium resource, it is necessary to recovery lithium from a low concentrated lithium-containing solution which can solve the shortage of lithium resources and avoid the waste of lithium. In this article, the lithium phosphate is recovered from lithium-containing solution with a concentration of 2 g/L after preparation of lithium carbonate. The results show that by the application of ultrasound, the lithium recovery rate can be increased. The concentration of lithium is less than 0.3 g/L after preparation of lithium phosphate. For lithium carbonate recovery by ultrasound, please refer to the full length article entitled “Lithium carbonate recovery from lithium-containing solution by ultrasound assisted precipitation”, https://doi.org/10.1016/j.ultsonch.2018.12.025 (Chunlong Zhao et al., 2019) [1].

**Table:** Specifications table

Subject area	Energy
More specific subject area	Renewable Energy, Sustainability and the Environment
Type of data	Table, image (XRD and SEM), figure
How data was acquired	ICP-OES (iCAP 6300, Radial, Thermo Scientific), X-ray diffraction spectrometer (X’pert PRO, PANalytical) and mineral liberation analyzer (MLA 250, FEI) which is equipped with an energy dispersive spectrometer (EDS, EDAX GenesisSiLi) and a scanning electron microscope (SEM, JEOL JSM-7610F),
Data format	Raw, Analyzed
Experimental factors	Recovery of lithium phosphate by using ultrasound under the following conditions: reaction temperature of 333 K, agitation speed of 400 rpm, reaction time of 35 min, ultrasound power of 150 W, solid Na_3_PO_4_, Na^+^ to Li^+^ of 1
Experimental features	Preparation of lithium phosphate
Data source location	Beijing Engineering Research Center of Process Pollution Control, National Engineering Laboratory for Hydrometallurgical Cleaner Production & Technology, Chinese Academy of Sciences, Beijing 100190, China
Data accessibility	Data are presented in this article
Related research article	Chunlong Zhao et al. “Lithium carbonate recovery from lithium-containing solution by ultrasound assisted precipitation”, Ultrason. Sonochem. 52, 2019, 484–492 [1]. https://doi.org/10.1016/j.ultsonch.2018.12.025

**Table:** 

**Value of the data** • This data set enriched the globe lithium recovery from lithium-containing solution by ultrasound assisted precipitation. • Data provides information about recovery lithium from a low-concentrated lithium-containing solution. • This data can be used to develop a new way to recovery lithium from a low-concentrated lithium containing waste solution.

## 1 Data

**Fig. 1:**
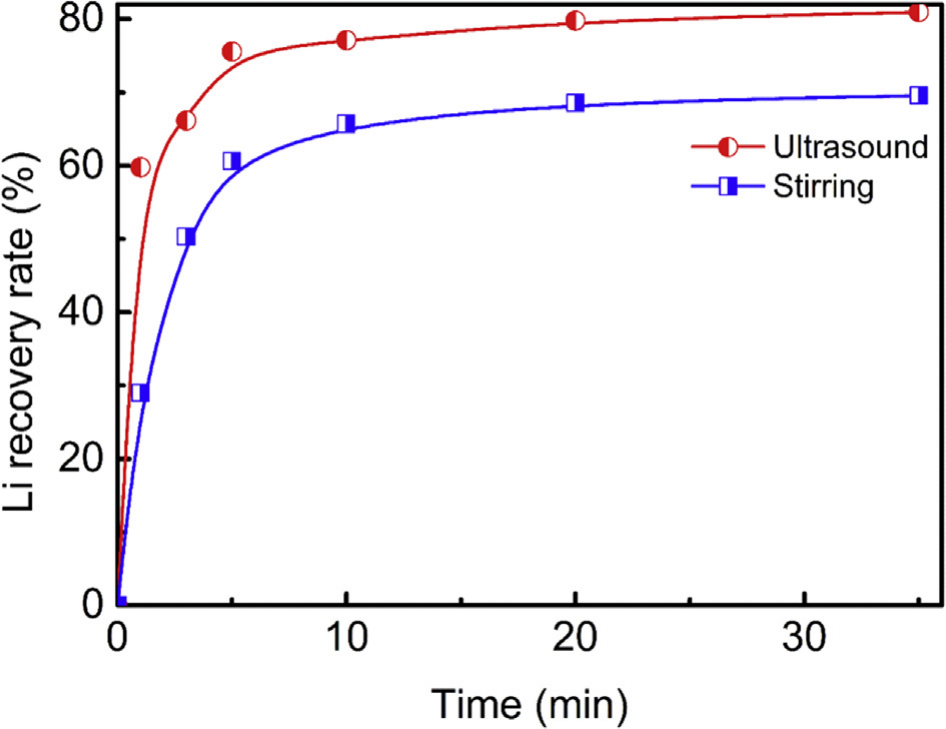
Effect of ultrasound on the crystallization time of Li_2_CO_3_ (ultrasound power of 150 W, solid Na_2_CO_3_, Na^+^ to Li^+^ of 1, initial lithium concentration of 10 g/L, at 343 K for 35 min).

**Fig. 2:**
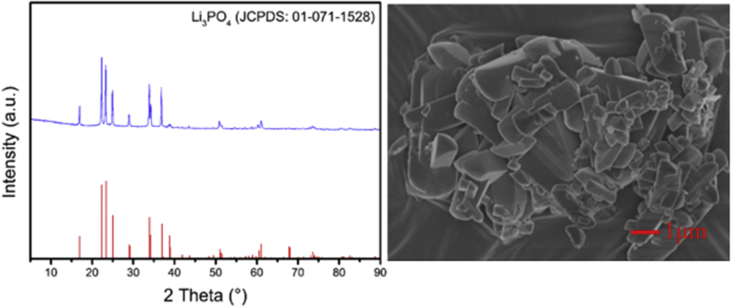
The XRD pattern and SEM image of Li_3_PO_4_.

Data shown in this article are related to the research article entitled “Lithium carbonate recovery from lithium-containing solution by ultrasound assisted precipitation” [1]. The initial content of the leachate of cathode scrap before removing impurity ions is presented in Table 1. The effect of ultrasound on the crystallization time of Li_2_CO_3_ is provided in Fig. 1. Table 2 is the mass fraction of metals in the precipitated Li_3_PO_4_. Fig. 2. Is the XRD pattern and SEM image of Li_3_PO_4_.

**Table 1:** The leachate of cathode scrap before removing impurities.

Metal	Li	Ni	Mn	Co	Al
Concentration (g/L)	10 ± 0.5	11 ± 0.2	10.5 ± 0.2	3.2 ± 0.2	5 ± 0.2

**Table 2:** Mass fraction of metals in the precipitated Li_3_PO_4_.

Content	Li_3_PO_4_	Na_2_O	NiO	MnO	Co_2_O_3_	Al_2_O_3_
Composition (wt %)	95.5705	4.4041	0.0046	0.0028	0.0076	0.0104

## 2 Experimental design, materials and methods

The lithium concentration of filtrate (FI) after recovery lithium carbonate was 2 ± 0.2 g/L, 100 ml FI was added into the reactor (Fig. 1 in Ref. [1]) at 60 °C and the ultrasonic probe was immersed into the FI. The stoichiometric solid sodium phosphate (Na_3_PO_4_) was added to the FI at one time and the ultrasonic instrument was also opened simultaneously. After the reaction was completed, the FI was filtered immediately and the precipitant was dried in an oven for 24 h at 80 °C, the Li_3_PO_4_ can be obtained. The lithium recovery rate can also be calculated from Eq. (1).

(1) $$I_{R}=\;1-\frac{C_{t}V_{t}}{C_{0}V_{0}}$$

where, *I*_*R*_ refers to the lithium recovery rate, *C*_*t*_, *C*_*0*_ refers to the concentration of lithium at time t and initial solution (g/L), *V*_*t*_ and *V*_*0*_ refer to the volume of solution at time t and initial solution (ml), respectively.

The initial content of leachate of cathode scrap before removing impurities was illustrated in Table and the effect of ultrasound on the crystallization time of Li_2_CO_3_ was given in Fig. 1. After precipitating Li_2_CO_3_ and filtration, the FI was obtained with concentration of 2 ± 0.2 g/L. Subsequently, the Li_3_PO_4_ can be obtained with recovery rate of 85.1% by adding stoichiometry (100%) Na_3_PO_4_ which is insoluble in water [2] and the parameters were chosen as follows: solid Na_3_PO_4_, sodium to lithium of 1, at 333 K for 35 min. The XRD pattern of the obtained Li_3_PO_4_ is shown in Fig. 2. It agrees well with the standard pattern peaks of Li_3_PO_4_ (JCPDS: 01-071-1528). To accurately calculate the purity of Li_3_PO_4_, the precipitated Li_3_PO_4_ was further dissolved by aqua regia and its mass fraction of metals was measured by ICP-OES. The results were shown in Table 2 and the mass fraction of Li_3_PO_4_ is accounted for 95.57%. The SEM image of the precipitated Li_3_PO_4_ is illustrated in Fig. 2, and it can be observed that the precipitated Li_3_PO_4_ was presented as rod-like crystal.
